# The utility of image-enhanced endoscopy and Lugol’s for the assessment of esophageal squamous carcinoma

**DOI:** 10.1016/j.vgie.2021.10.004

**Published:** 2021-11-26

**Authors:** Douglas Motomura, David Hurlbut, Wiley Chung, Robert Bechara

**Affiliations:** Hotel Dieu Hospital,Queens University, Kingston Health Sciences Center, Kingston, Ontario, Canada

**Keywords:** ESCC, esophageal squamous cell carcinoma, ESD, endoscopic submucosal dissection, HGD, high-grade dysplasia, SCC, squamous cell carcinoma

## Abstract

Video 1 This is a case of a patient with esophageal squamous cell carcinoma who underwent endoscopic submucosal dissection and subsequent esophagectomy. The endoscopic findings using imaged endoscopy, magnification, and Lugol’s are demonstrated. There is a demonstration of endoscopic and pathological correlation in addition to the emphasis on a patient-centered multidisciplinary approach to esophageal neoplasia and the diagnostic utility of endoscopic submucosal dissection.

This is a case of a patient with esophageal squamous cell carcinoma who underwent endoscopic submucosal dissection and subsequent esophagectomy. The endoscopic findings using imaged endoscopy, magnification, and Lugol’s are demonstrated. There is a demonstration of endoscopic and pathological correlation in addition to the emphasis on a patient-centered multidisciplinary approach to esophageal neoplasia and the diagnostic utility of endoscopic submucosal dissection.

## Introduction

Esophageal squamous cell carcinoma (ESCC) is the most commonly diagnosed esophageal cancer.^1^ Previously, diagnoses in the late stages of disease led to a poor prognosis and limited treatment. However, advancements in optical diagnosis and resection techniques have allowed for improved detection, characterization, and therapy. We present a case that highlights the utility of image-enhanced endoscopy and Lugol’s in the assessment of ESCC (Video 1, available online at www.giejournal.org).

## Case report

A 51-year-old man was referred for assessment of an esophageal squamous lesion in the proximal esophagus. The referral biopsies showed high-grade dysplasia (HGD), and staging CT-positron emission tomography did not demonstrate evidence of metastatic disease.

**Figure 1:**
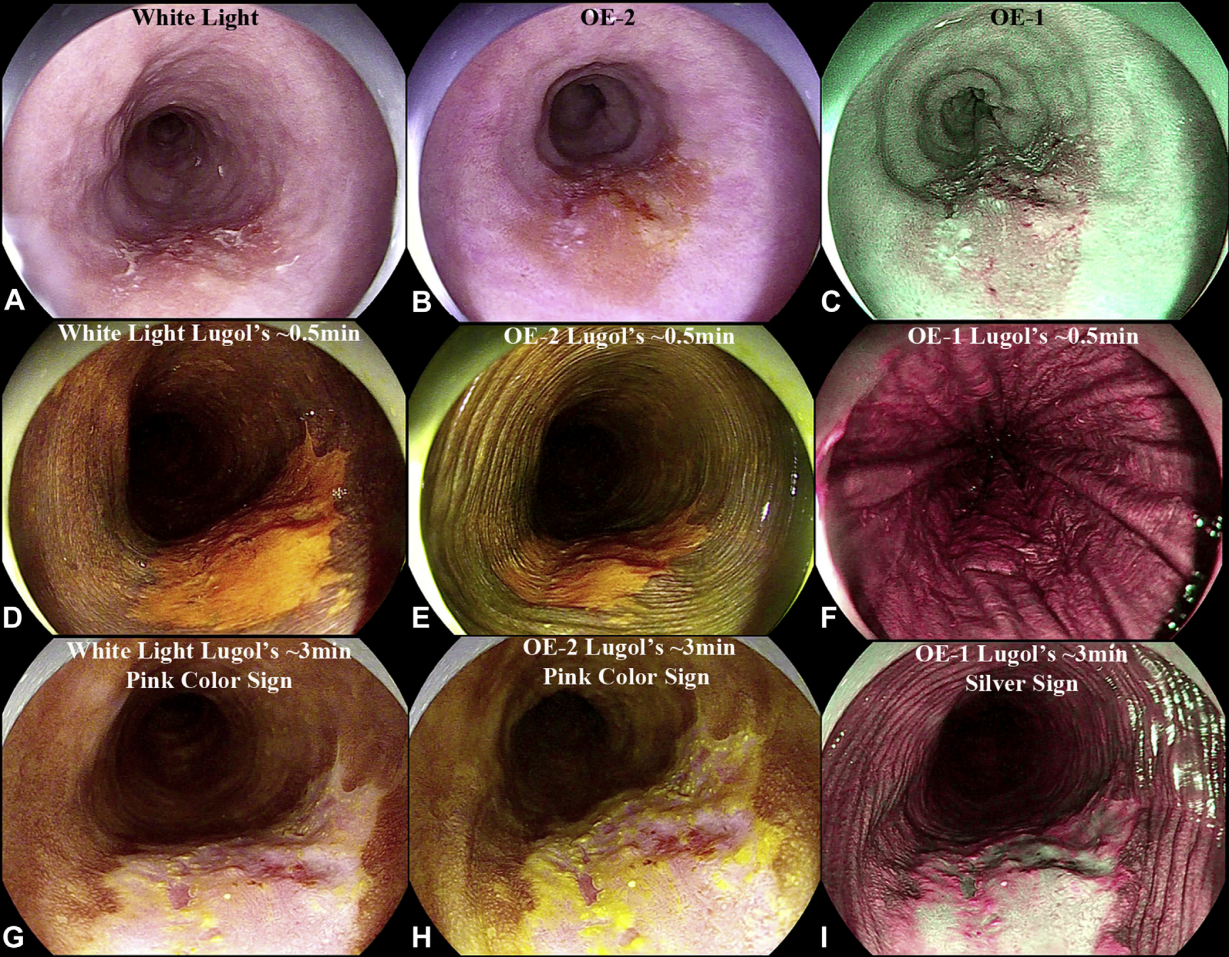
Macroscopic findings of the squamous cell carcinoma. **A,** White light examination (WLE). **B,** Optical enhancement mode 2 (OE-2). **C,** Optical enhancement mode 1 (OE-1). Thirty seconds after 1.5% Lugol’s. **D,** WLE. **E,** OE-2. **F,** OE1. Three minutes after 1.5% of Lugol’s. **G,** WLE. **H,** OE-2. **I,** OE-1.

**Figure 2:**
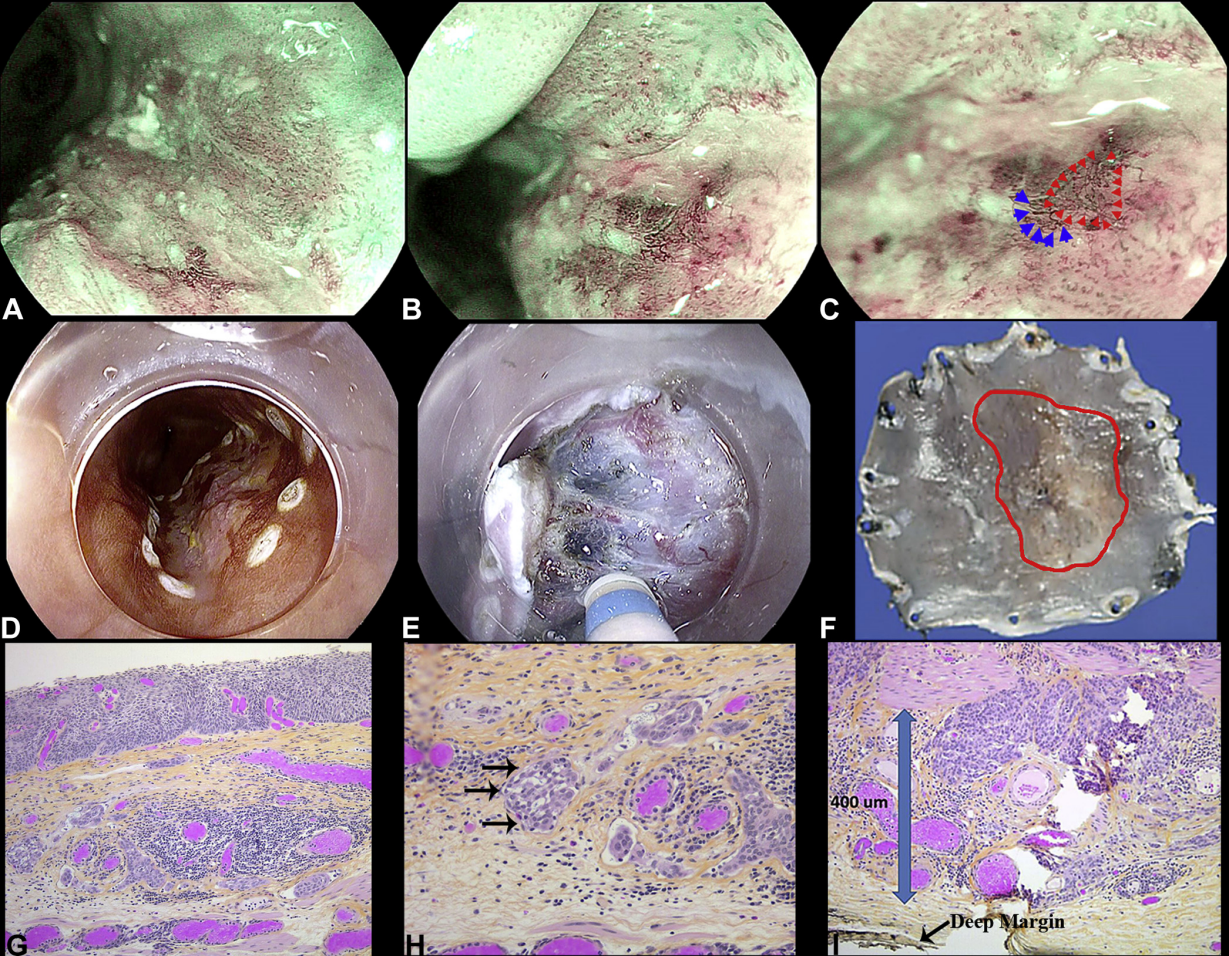
Magnification and resection of the squamous cell carcinoma. **A,** Optical enhancement mode 1 (OE-1) low magnification. **B,** OE-1 medium magnification. **C,** OE-1 high magnification (*blue arrows* indicate B2 vessels and *red arrow heads* indicates reticular pattern vessels). **D,** Marking. **E,** Submucosal dissection. **F,** Gross specimen with depressed area with B2 vessels outlined in red. **G,** In situ squamous cell carcinoma. Lymphovascular invasion present within underlying lamina propria with hematoxylin phloxine saffron stain, orig. mag. ×50. **H,** Tumor in endothelial-lined spaces, orig. mag. ×200. **I,** SM2 disease, orig. mag. ×50.

Examination using white light and optical enhancement modes revealed a Paris IIa + IIc lesion (Fig. 1A-C). Chromoendoscopy with 1.5% Lugol’s iodine was performed. At 30 seconds, the lesion is clearly delineated from the surrounding mucosa as a Lugol’s voiding lesion (Fig. 1D-F). At 3 minutes, the pink color sign and silver sign are apparent, indicative of at least HGD (Fig. 1G-I). Magnification using optical enhancement mode-1, which uses light wavelengths 415 nm + 540 nm, allowed for characterization of the microvasculature, demonstrating mildly dilated vessels lacking loop formation (B2 vessels^2^). A reticular vascular pattern was also noted (Fig. 2A-C).

After discussion with the patient and presentation at multidisciplinary esophageal tumor rounds, the decision was made to proceed with a diagnostic and potentially curative endoscopic submucosal dissection (ESD). The possibility of esophagectomy was discussed and planned to ensure that the ESD would not delay surgical management if required. In accordance with the patient’s goals, this approach gave the greatest likelihood of avoiding surgery.

**Figure 3:**
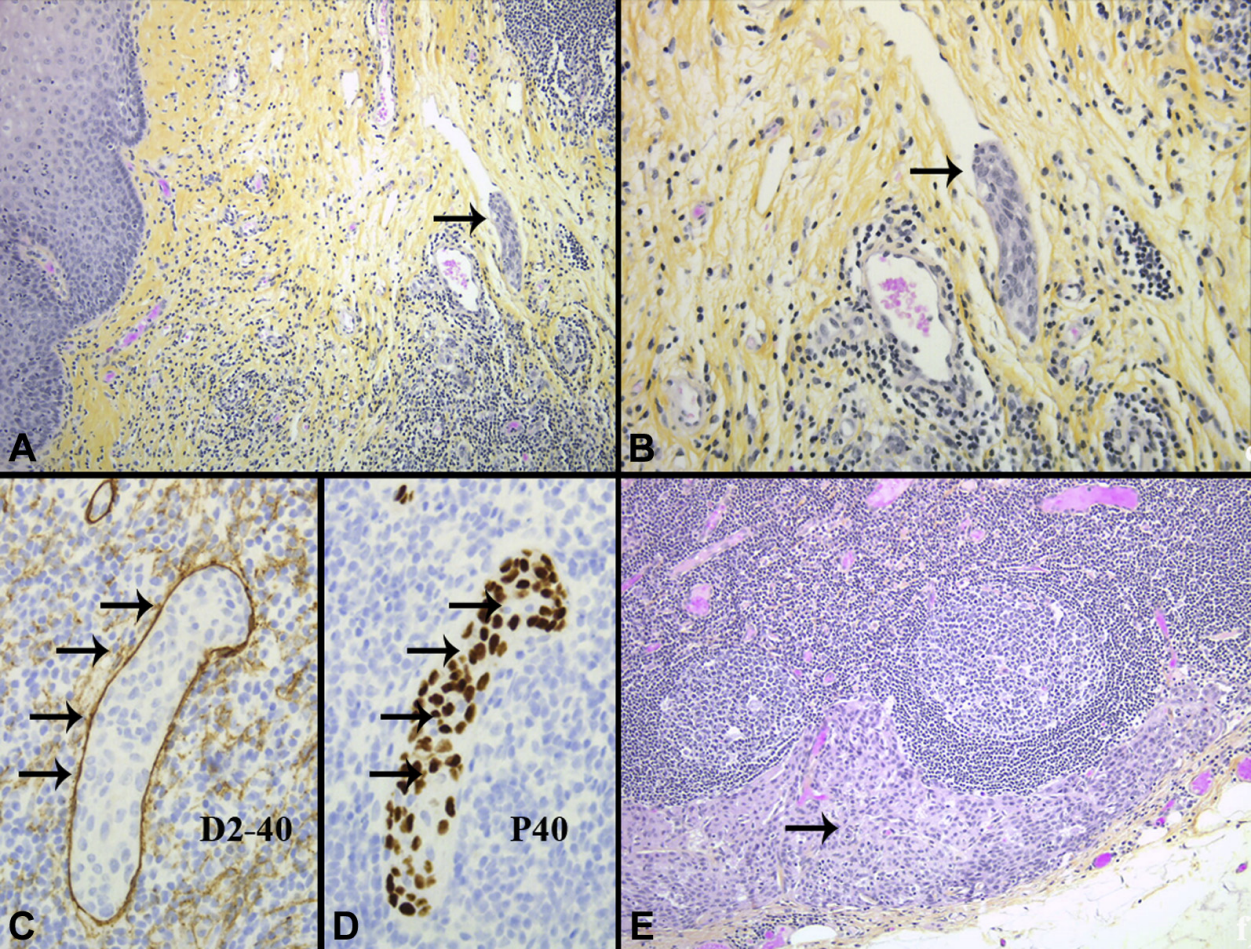
Esophagectomy intralymphatic squamous cell carcinoma (SCC) at proximal anastomosis donut. Proximal resection margin donut with lymphovascular invasion by SCC. **A,** Orig. mag. ×50. **B,** Orig. mag. ×100. Lymphovascular invasion by SCC, orig. mag. ×200. **C,** D2-40—highlights lymphatic space. **D,** P40—stains SCC tumor cell nuclei within the lymphatic space. **E,** Metastatic SCC in lymph node, orig. mag. ×100.

The lesion was removed en bloc during a 39-minute procedure (Fig. 2D-F). Final histology results revealed squamous cell carcinoma (SCC) with SM2 invasion (T1b), moderate-poor differentiation, and lymphovascular invasion (Fig. 2G-I). The resection margins were clear (R0). Because of the high-risk features (T1b, poor-differentiation, lymphovascular invasion), the patient underwent esophagectomy. The pathology revealed clear margins at the site of the ESD resection, 1 positive lymph node (N1), and SCC intravascularly (Fig. 3).

## Discussion

Based on the Paris classification, 50% of lesions with IIa + IIc morphology have at least SM2 involvement.^3^ Chromoendoscopy with iodine staining is a useful technique for detection of ESCC. The sensitivity and specificity of a “pink color sign” for HGD or worse after Lugol’s staining is reported to be 88% and 95%, respectively.^4^ When optical enhancement mode-1 (which contains light wavelengths 415 nm and 540 nm) is used, the silver sign is demonstrated, which is considered equivalent.^5^

By examining the intrapapillary capillary loops using magnifying endoscopy, depth of invasion can be estimated according to the Japanese Esophageal Society classification.^2^ In our case, B2 vessels were seen in the area of depression. These are suggested to have 75% sensitivity and 96% specificity for M3 (carcinoma to the muscularis mucosa) to Sm-1 (submucosal invasion to 200 μm) invasion, which would be a relative indication for ESD. Normal capillary loops are thin and maintained in a loop formation. By contrast, B1 vessels are dilated but retain loop configuration and indicate mucosal limited disease. B3 vessels are severely dilated and are associated with cancer invading to Sm-2 or deeper.^2^

In our case, other than the clearly depressed area, there were no other high-risk features to suggest deeper invasion, such as poor tissue pliability or nodular protrusion.^6^ Additionally, the poor differentiation may have been suggested by the reticular pattern of vessels.^2^ Nonetheless, because the ESD would provide definitive histology, would not delay surgery if needed, and would provide the patient the potential of avoiding an esophagectomy, it was decided in discussion with the patient and at multidisciplinary tumor boards to proceed with ESD as the first step.

Thus, careful examination with enhanced endoscopy and Lugol’s can provide vital and incremental pieces of information during the endoscopic assessment of ESCC. The use of this information allows for a more informed, comprehensive, and patient-centered treatment plan. This case also highlights the importance of multidisciplinary discussion and potential surgical planning in the management of early ESCC.

## Disclosure

*Dr Bechara is a consultant for Olympus and Pentax. All other authors disclosed no financial relationships.*

## Supplementary data

Video 1 This is a case of a patient with esophageal squamous cell carcinoma who underwent endoscopic submucosal dissection and subsequent esophagectomy. The endoscopic findings using imaged endoscopy, magnification, and Lugol’s are demonstrated. There is a demonstration of endoscopic and pathological correlation in addition to the emphasis on a patient-centered multidisciplinary approach to esophageal neoplasia and the diagnostic utility of endoscopic submucosal dissection.
